# Scalpel edge roughness affects post-transection peripheral nerve regeneration

**DOI:** 10.1016/j.sopen.2020.11.002

**Published:** 2020-11-18

**Authors:** Hannes Prescher, Michelle X. Ling, Victoria Bigdelle, Clifford L. Spiro, Raphael C. Lee

**Affiliations:** Laboratory for Molecular Regeneration, Section of Plastic and Reconstructive Surgery, The University of Chicago, Chicago, IL 60637

## Abstract

**Background:**

Gentle and precise tissue dissection reduces collateral tissue damage and preserves its structural quality for optimizing healing. This is particularly true for peripheral nerve neurorrhaphy. Axon regeneration kinetics across the repair is dependent on the amount of intraneural fibrosis. The purpose of this study was to determine whether scalpel blade smoothness was a deterministic factor in the kinetics of postneurorrhaphy peripheral axon regeneration.

**Methods:**

Scalpel transection of the saphenous nerve was performed in 18 female Hartley guinea pigs either by a standard #15 stainless steel scalpel blade or a highly polished version of the same blade. Compound nerve action potential recordings and histochemical assay of neurofilament density proximal and 1 cm distal to the site of nerve transection were quantified postneurorrhaphy at postoperative weeks 5, 9, and 12.

**Results:**

There was no action potential transmission observed in the distal axons immediately after neurorrhaphy. A substantial acceleration of axonal conduction recovery was observed in nerves transected with polished scalpel blades observed by high compound nerve action potential amplitudes at postneurorrhaphy weeks 5 and 9 (P < .05). In addition, an increased recovery of intra-axonal neurofilament density in nerves transected with polished scalpel blades was observed by postoperative week 5 (P < .05).

**Conclusion:**

The quality of the scalpel blade is an important determinate of postsurgical healing. Gentle handling of tissue matters.

## Highlights

• Precise nerve resection reduces lateral axonal injury.
• Collateral nerve damage increases interneural fibrosis that slows regeneration.
• Smoother scalpel blades result in faster structural and functional nerve recovery.

## INTRODUCTION

Atraumatic manipulation of soft tissues during surgical dissection is a central principle of proper surgical technique. Any structural disruption of the wound boundaries triggers a local inflammatory and healing response that results in fibrosis and contracture [1]. Of course, since the magnitude of the healing response must scale according to the extent of injury, more tissue injury results in more local tissue inflammation, pain, and fibrosis. [[2], [3]] For this reason, mechanical crush injury from rough surgical manipulation or use of dull dissection instruments are problematic; the trauma is spread over a larger surface area than needed. It stands to reason that minimizing the collateral tissue crush component of surgical dissection by using smoother and polished surface-finished surgical instruments, including scalpel blades, reduces postsurgical tissue inflammation and fibrosis.

This is especially true for peripheral nerve surgery. Peripheral nerve injury is followed by a multicellular response that results in both nerve regeneration as well as intra- and perineural fibrosis. Nerve injury repair is initiated by structural disruption to the plasmalemma membrane, which sets off a cascade of events leading to sealing of the axon proximal to the injury and distal necrosis [4,5]. Distal axonal degeneration is followed by degradation of the myelin sheath surrounding the axon distal to the site of injury [6]. Schwann cells follow and align into tubes, which guide regenerating nerve fibers from the proximal nerve segment [7,8]. However, the success of these regeneration processes is impaired by excess fibrosis.

When a scalpel blade is used to transect a nerve for repair or grafting, the amount of nerve injury depends on the quality of the blade. The standard stainless-steel surgical blades commonly used today are manufactured using a grinding mechanism with a diamond embedded grinding wheel. However, contact with these abrasive materials creates contour irregularities in the blade as the embedded diamonds plough into the metal. When applied to soft tissues, these irregularities lead to nonuniform distribution of the deformational forces of the blade. This creates shearing of the tissue and a local crush injury. A metal polishing process was used to minimize surface features along the blade surfaces and edge [9], thereby increasing blade smoothness. This refinement consists of a variant of chemical mechanical planarization (CMP), a process widely practiced in the manufacture of advanced semiconductors used in memory and processing computer chips. With CMP, a suite of chemistries reacts with and softens various metal and ceramic surfaces, which is followed by the mechanical removal of the softened surface with a combination of a polishing tool, pad, and accompanying microscopic particles. This consistently produces surfaces of near-atomic perfection.

Fundamentally, the hypothesis of this project is that more polished scalpel surfaces and edges should reduce local tissue trauma and facilitate healing in contrast to the ragged edges currently employed. Thus, the purpose of this study was to determine if the scalpel edge smoothness has any impact on the rate of structural and functional nerve regeneration following nerve transection.

## METHODS

### Scalpel Blades Used for the Study

**Fig 1:**
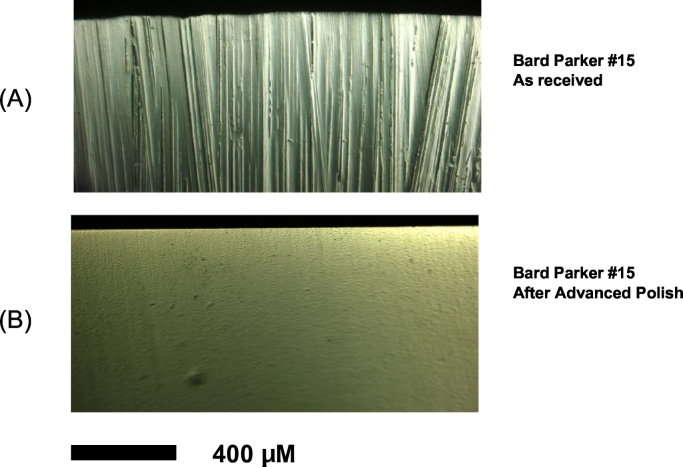
Optical photomicrographs demonstrating the topographical difference between a standard scalpel blade (A) and polished blade (B). Bar scale represents 400 μmol/L.

The experimental blades used in this study were produced using the polishing process described above [9]. Polished blades were produced from off-the-shelf Bard-Parker #15 stainless steel slot blades. All control blades were standard, unprocessed Bard-Parker #15 stainless steel slot blades from the same lot as the polished blades. Optical photomicrographs of each blade demonstrate the difference in topography (Fig 1). Keyence optical interferometry was used to analyze the average smoothness (ie, degree of uniformity of the surface topography) of the respective blade surfaces. Measurements were subsequently confirmed by Dektak contact profilometry using a diamond stylus.

### Animals

Female Hartley guinea pigs (Charles River Labs, Wilmington, MA) weighing 400 ± 20 g were used in all experiments. A total of 18 animals were used in the experiments. Anesthesia was induced by intraperitoneal injection of ketamine/xylazine and maintained with titrated isoflurane gas (3% in 1.5-L/min oxygen flow) administered via face mask. Vital signs were monitored every 15 minutes throughout the procedure. All animals received analgesia postoperatively consisting of a subcutaneous dose of buprenorphine (0.05 mg/kg). The animals were euthanized at the end of the study with an overdose (20 mg/kg) of xylazine.

The protocol was approved by the University of Chicago Institutional Animal Care and Use Committee. Each animal was housed in an individual cage. Animals were cared for in the standard procedure of the central housing facility following the guidelines of the National Institutes of Health described in *The Guide for the Care and Use of Laboratory Animals* [11].

### Saphenous Nerve Injury and Repair

All surgical procedures were performed under a standard Weil surgical operating microscope by a single surgeon (RCL). Nerve transection and repair were performed under general anesthesia as described above. Once anesthetic depth and lack of response to skin and toe pinch were confirmed, the surgical procedure was performed.

A standardized surgical approach was used for each animal. A sterile surgical field was maintained, and a skin incision was made over the left medial vastus muscle. The dissection was carried through the muscle in a muscle-sparing fashion to expose the left saphenous nerve. A firm, plastic strip was placed under the exposed nerve, and two 10-0 nylon alignment sutures were run beneath the epineurium longitudinally on opposite sides of the nerve. The nerve was wrapped in a thin polyethylene sheeting (ie, Saran Wrap) to keep its shape. A perpendicular transection through the wrapped nerve segment in the proximal third of a 2-cm segment of nerve was then performed in a single pass using either the control or sharpened surgical scalpel blade. Scalpels used for the nerve transections were only used for the nerve transection and not used for any other part of the surgical procedure. The surgeon was blinded to the type of scalpel used, which was prospectively randomized to either the standard (*n* = 9) or polished blade (*n* = 9).

Post-transection neurorrhaphy was carried out using two 10-0 nylon interrupted epineural sutures. The remaining epineurium was reconnected and sealed using Dermabond adhesive (Ethicon Inc, Somerville, NJ). This technique has been shown to be as effective as microsuture repair in functional outcomes following nerve injury [12]. The skin incision was then closed in 2 layers. Animals in the control and experimental groups were then subdivided into 3 groups of 3 animals each to be examined at postoperative weeks 5, 9, and 12, respectively.

### Measurement of Compound Nerve Action Potential

The compound nerve action potential (CNAP) amplitude values were obtained using direct electrode recordings on the surface of the saphenous nerve. Dantec Counterpoint II neurodiagnostic system was used for action potential stimulation and recording. Two monopolar stimulation electrodes (Teca Monopolar) separated by 2 mm were placed on the surface of the saphenous nerve 0.5 cm proximal to the transection site. A concentric recording electrode (Teca Elite) was placed 1 cm distal to the transection site.

The current source stimulator was used to stimulate the CNAP. The concentric electrode was attached to high-impedance FET amplifiers on the Dantec head stage. The stimulus amplitude was adjusted to achieve the maximal amplitude of the nerve recording. Stimulation intensities of 0.4 to 0.6 mA were needed with a duration of 0.1 millisecond. CNAP measurements were obtained from the left saphenous nerve at 5, 9, and 12 weeks postoperatively. The CNAP was also measured for the right saphenous nerve as a nonoperative control at the same time intervals.

### Histology-Immunohistochemistry

Recovery at a point 0.5 cm proximal and distal to the injury was monitored by immunohistochemistry (IHC**)** of paraffin-bound biopsies of the saphenous nerve axon. A monoclonal antineurofilament protein was utilized to identify neurofilament-positive cells (DAKO North America, Inc, Santa Clarita, CA). Optimal antibody dilution was determined by titration. Dako neurofilament antibody was labeled with avidin-biotinylated horseradish peroxidase complex (ABC) according to manufacturer’s instructions. The optical density of the stained sections was then quantified in a blinded fashion with an automated cellular imaging system (Chromavision Medical Systems ACIS III, San Juan Capistrano, CA). Optical density is reported as a percentage of the total image area that contains the dye. Animals for each group were analyzed on postoperative weeks 5, 9, and 12. IHC analysis was performed for the left saphenous nerve and the right saphenous nerve for each animal.

### Electron Microscopy

Electron microscopy (EM) was used to analyze the degree of myelination at a point 0.5 cm proximal and distal to the transected nerve. A small segment of nerve was fixed in 2.5% glutaraldehyde followed by 1% osmium tetroxide. It was then embedded in araldite and submitted for EM. Animals for each group were analyzed on postoperative weeks 5, 9, and 12, and EM analysis was performed on both the left saphenous nerve and the right saphenous nerve. All immunostaining, electron microscopy, and analysis were performed in the pathology core facility at the University of Chicago.

### Statistical Analysis

All measurements were assumed to be independent within groups. The magnitude of the action potentials from the transected and repaired saphenous nerves was normalized to the uninjured (control) saphenous nerve in the same animal. This was done to reduce animal-to-animal variation. Values for each animal are reported as a percentage of the contralateral control. Analyses of differences among the means were performed using analysis of variance. The data were log transformed to achieve a normal distribution, and a Tukey test was performed to determine significant differences in the expected means between polished and control blade groups.

## RESULTS

### Animals

All of the guinea pigs survived the surgery and were maintained without signs of pain or distress. Two animals were lost early in the experiment because of self-inflicted injury to their foot on the side of the nerve transection. These 2 animals were replaced with animals that did not sustain injuries over the course of the experiment.

### Surgical Blade

Analysis of the blade surfaces using Keyence optical interferometry revealed a root-mean-square roughness of 481 nm for the control blades compared to 25 nm for the polished blades (*P* < .05). Dektak contact profilometry using a diamond stylus confirmed an average roughness of 28.2 nm for the polished blades. The maximum peak-to-valley roughness averaged 4.1 μm for the control blades compared to 0.20 μm for the polished blades (*P* < .05). Optical images of the control and polished surgical blades are shown in Fig 1.

### Compound Nerve Action Potential

**Fig 2:**
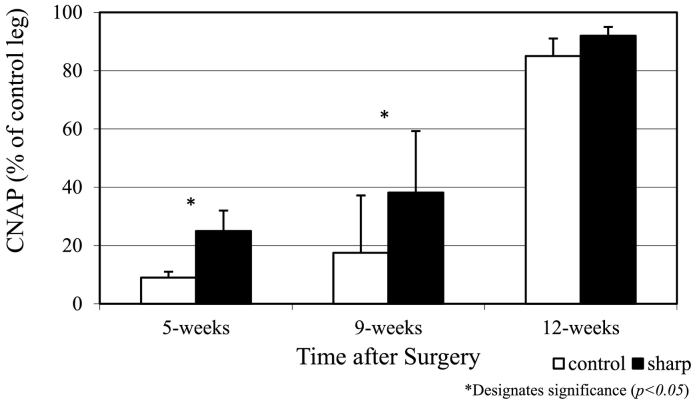
Recovery after nerve transection as demonstrated by recovery of the compound nerve action potential. Data are expressed as percentage of contralateral control nerve as a function of time after nerve transection. An improvement in conduction was seen as early as 5 weeks postoperatively with the polished blade and did not become equivalent until 12 weeks postoperatively (*P* < .05).

A significant improvement in the rate of axon regeneration into the distal neural sheath, as reflected in the CNAP amplitudes, was observed for nerves transected with the polished blade versus those transected with standard scalpel blades. The mean CNAP amplitude (millivolts) for the polished blade group was 25.0% ± 7.0% compared to 9.0% ± 2.0% for the control blades at 5 weeks post-transection (*P* < .05; Table 1, Fig 2). Similarly, at 9 weeks postneurorrhaphy, the CNAP amplitude was 38.2% ± 21.0% for the polished blade and 17.5% ± 20.0% for standard blades (*P <* .05). At 12 weeks after surgery, there was no significant difference in the mean CNAP measured between nerves transected with the polished versus the control blades (92.0% ± 3.0% vs 85.0% ± 6.0%; *P* > .1).

**Table 1:** Immunohistochemical label density reflecting neurofilament density measured from nerve segments proximal and distal to the transected guinea pig saphenous nerve

*Time*	*CNAP magnitude*		*IHC (proximal to injury)*		*IHC (distal to injury)*	
	*Polished*	*Control*	*Polished*	*Control*	*Polished*	*Control*
5 wk	25.0 ± 7.0	9.0 ± 2.0⁎	82.4 ± 4.0	79.6 ± 3.5	41.7 ± 12.9	21.7 ± 11.1⁎
9 wk	38.2 ± 21.0	17.5 ± 20.0⁎	79.1 ± 12.4	81.1 ± 6.7	45.4 ± 16.9	37.9 ± 4.3
12 wk	92.0 ± 3.0	85.0 ± 6.0	79.4 ± 10.3	76.9 ± 9.6	47.0 ± 9.1	40.1 ± 11.9

All data are presented as percentage of contralateral control.

⁎ Designates significance (*P* < .05).

### Immunohistochemical Assay of Axon Neurofilament Density

**Fig 3:**
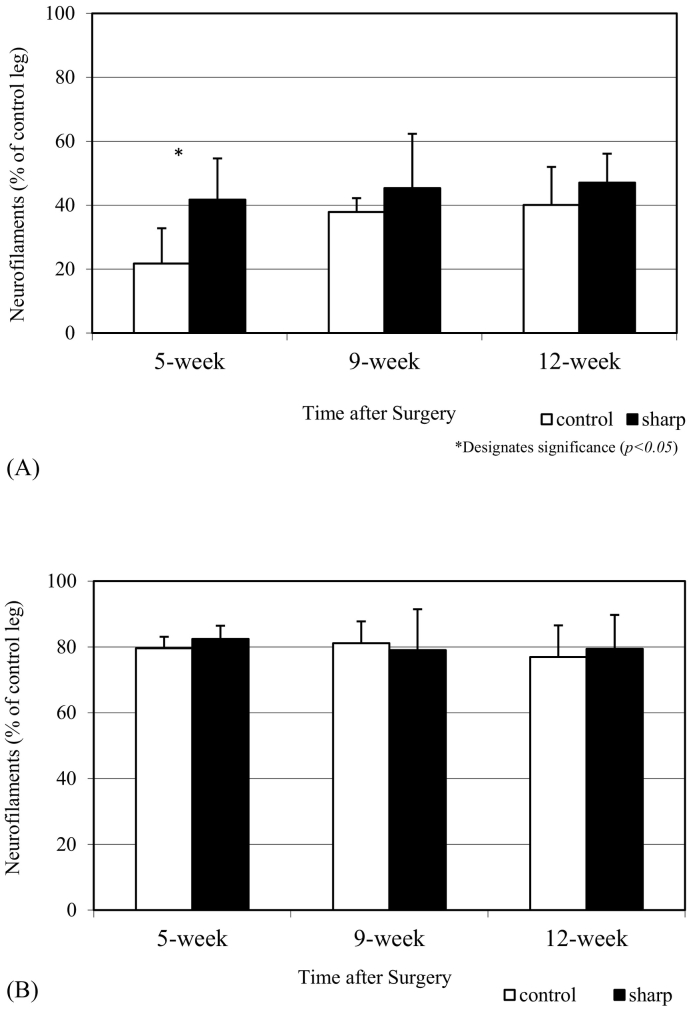
(A) An increased density of nerve fibers *distal* to the transection was demonstrated at 5 weeks postoperatively for saphenous nerves transected with polished blades (*P <* .05). No significant difference was seen at other time intervals (*P* > .05). (B) No difference in the density of nerve fibers was seen *proximal* to the transection at any point postoperatively.

The segment of axon distal to the site of injury in the polished blade group demonstrated a significant increase in neurofilament density at 5 weeks postoperatively. The calculated optical density for the polished blade group was 41.7% ± 12.9% compared to 21.7% ± 11.1% for the control standard blade group (*P <* .05; Table 1). No significant difference was found at 9 weeks (45.4% ± 16.9% vs 37.9% ± 4.3%; *P* > .05) and 12 weeks (47.0% ± 9.1% vs 40.1% ± 11.9%; *P* > .05) postoperatively. No difference was seen in the neurofilament density in the segment of axon taken from proximal to the site of injury at 5 weeks (82.4% ± 4.0% vs 79.6% ± 3.5%; *P* > .05), 9 weeks (79.1% ± 12.4% vs 81.1% ± 6.7%; *P* > .05), or 12 weeks (79.4% ± 10.3% vs 76.9% ± 9.6%; *P* > .05) after injury (Table 1; Figs 3B and 5). There was no evidence of neuroma formation, and no Dermabond adhesive residue was observed between the 2 nerve endings.

### Microscopy

**Fig 4:**
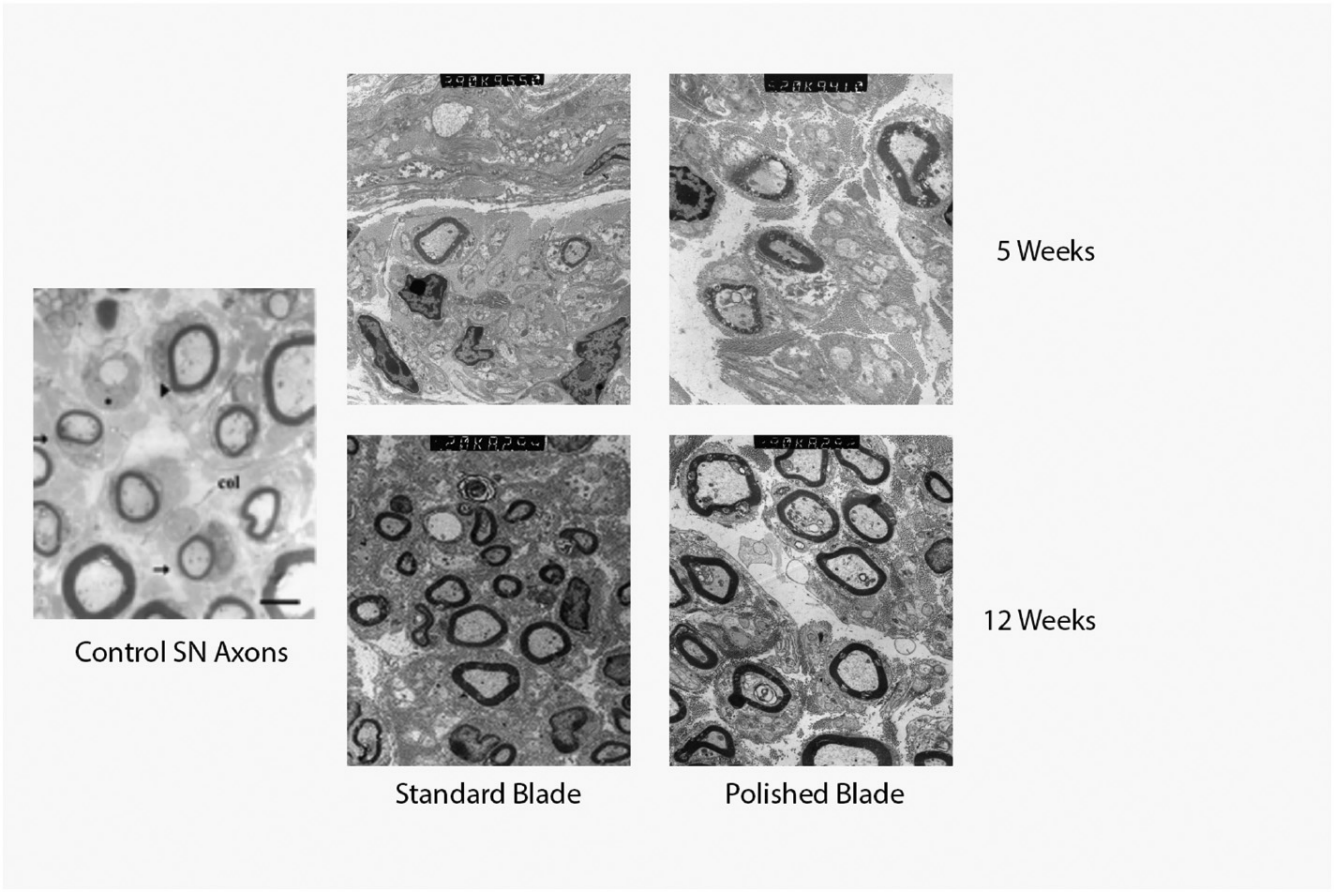
Representative EM images reveal a significant increase in myelination at 5 weeks after saphenous nerve (SN) transection with the polished blade (right column) compared to the standard blade (center column). At 12 weeks, there were no significant differences in myelination.

The segment of axon distal to the site of injury in the polished blade group demonstrated a significant increase in myelination at 5 weeks postoperatively (Fig 4). No difference was seen at 9 or 12 weeks postoperatively.

**Fig 5:**
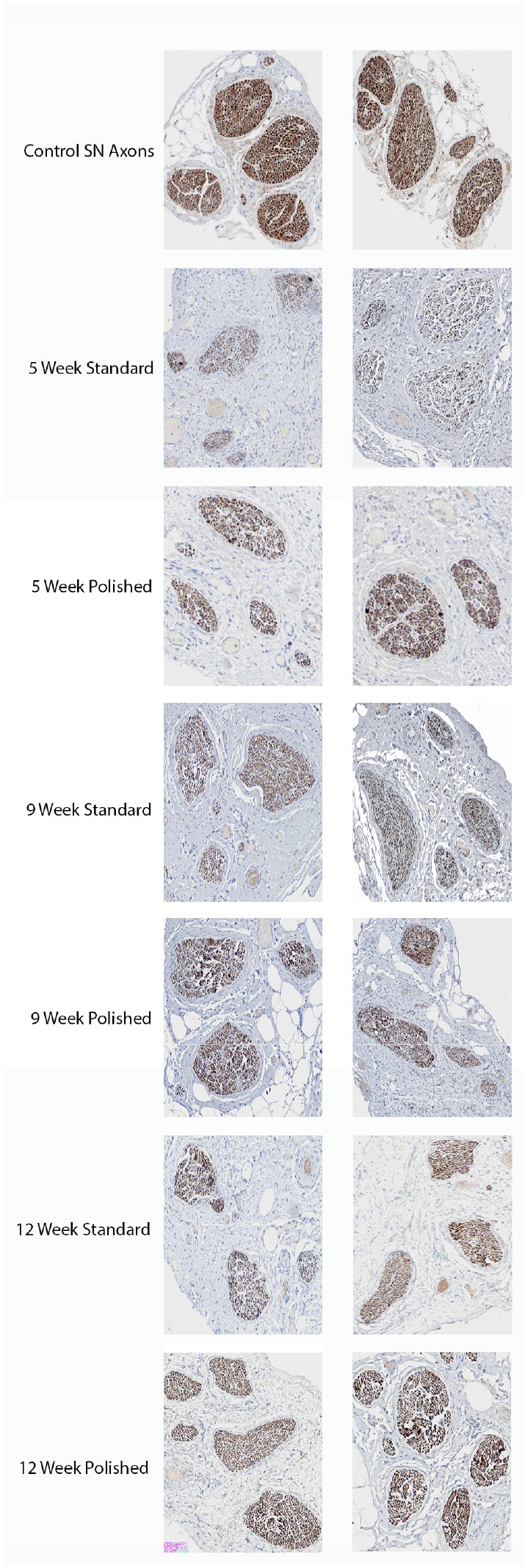
Nerve regeneration shown by IHC of neurofilaments at 5, 9, and 12 weeks postoperatively in saphenous nerve transected by polished blades versus standard blade.

## Discussion

The results of this study support the hypothesis that smoother scalpel blades can reduce local traumatic tissue injury and improve wound healing. Using a highly finished scalpel blade that has a 20-fold improvement in surface smoothness compared to the standard blade without additional polishing significantly accelerated structural and functional recovery after complete nerve transection. The enhanced scalpel blade was polished using a chemical-mechanical process that minimizes the surface contour deformities that can occur during scalpel blade manufacturing [9]. This technology is now becoming commercially available, increasing the feasibility of using a polished scalpel blade in clinical surgical practice.

Whenever tissue is cut with a surgical instrument, there will be divided tissue and a variable component of mechanically strained tissue injury. These iatrogenic injuries are particularly significant in peripheral nerve repair. Prior to neurorrhaphy, nerve ends are cut back sharply to optimize the precise realignment of individual nerve fascicles. While the crush damage component of the nerve transection may extend only minimally on each side of the cut, it can leave a clinically significant interposition of scar tissue, which can compromise the complex interaction between the regenerating proximal and distal nerve segments [13,14]. Reducing the volume of nerve damage results in less inflammation and scar tissue and thereby promotes nerve recovery [15,16].

Consequently, at 5 weeks after nerve transection, the CNAP conductivity of the nerve cut with the polished blade recovered to 25% of the contralateral unoperated side compared to only 9% recovery in the nerve cut with the standard blade. Likewise, the structural integrity of the distal nerve segment, as measured by neurofilament density and degree of myelination, at 5 weeks after transection recovered at twice the rate compared to the standard blade. Minimizing the crush element of the nerve transection by using a polished blade and accelerating CNAP conductance across the repair could improve functional outcomes [17]. Although there is some debate over how long sensory end organs remain viable, it is evident that early reinnervation improves functional return [18].

Clinically, these results are consistent with previous studies of peripheral nerve injuries. Previous studies have demonstrated reduced functional outcomes following digital nerve injuries with crush or avulsion mechanisms compared to more precise nerve transections [[19], [20], [21]]. Cadaveric studies have examined histologically the consequences of crush injuries compared to sharp transections and revealed significant disruption and fraying of nerve fascicles [22]. In the case of such crush injuries, nerves are debrided back sharply with a scalpel blade to healthy-appearing fascicles. Minimizing further crush damage to the nerve in the process is critical to optimize the healing potential and prevent neuroma formation [23].

Similar findings are seen in vascular anastomosis. Any shear or crush injury during adventitial stripping of the vessel in preparing it for anastomosis will trigger an inflammatory response and predispose the vessel to clotting, thus compromising the repair [[24], [25]]. The recent introduction of ultrasonic scalpels is predicated on the same concept, namely, to minimize collateral damage to surrounding structures during tissue manipulation and to increase the precision of tissue preparation [26].

However, repaired nerves rarely recover full preinjury function [10,[27], [28], [29]]. These results support this finding. At 12 weeks after transection, nerves cut with the standard and surface-finished blades achieved a CNAP amplitude of 85.0% and 92.0%, respectively, compared to the contralateral saphenous nerve. This is consistent with previous studies [30]. Interestingly, the rate of structural nerve recovery initially preceded functional recovery. At 5 weeks after transection, the nerve cut with the polished blade had recovered 41.7% of neurofilament density compared to only 25.0% of CNAP. Analysis at 9 and 12 weeks demonstrated a rapid increase in the rate of CNAP recovery, whereas the neurofilament density did not change significantly. At 12 weeks after transection, the CNAP had recovered to 92.0% of the contralateral control, whereas the neurofilament density remained at 47.0%. The repaired saphenous nerves were able to conduct nerve action potentials near preinjury magnitude in spite of significantly reduced number of neurofilaments. This indicates that the all fiber types are able to regenerate across the neurorrhaphy.

Nevertheless, this study has several limitations. CNAP amplitude 1 cm distal to the nerve repair was used to measure the number of regenerating axons in the saphenous nerve postneurorrhaphy. Although the correlation is correct, other factors like intraneural edema could influence CNAP amplitude. Although a single surgeon performed all nerve transections and repairs to eliminate interoperator variance, there is inexorable animal-to-animal variability in the transections and repair procedure which could have been influential. The low number of animals in each group also limits the statistical power of the study and the generalizability of our conclusions. Future studies will need to examine whether the use of highly polished blades will result in a difference in functional recovery from nerve healing. Also, it would be interesting to know if the use of polished surgical blades reduced granulation tissue and scar formation.

The goal of tissue handling is not to eliminate all iatrogenic injury. Any manipulation of tissue with a scalpel blade invariably produces an element of crush damage. However, by optimizing the surface finish of the blade, we can minimize the area of tissue damage. We have shown that using highly polished blades can facilitate more rapid structural and functional recovery following peripheral nerve transection in an established guinea pig model.

## Author Contribution

HP, MXL, VB, and RCL contributed to the design and implementation of the research, analysis of results, and writing of the manuscript. CLS contributed to the writing of the manuscript.

## Conflict of Interest

Dr Cliff Spiro is CTO of Entrepix Medical and a former employee of Cabot Microelectronics. For the remaining authors, none were declared.

## Funding Source

This work was supported by a grant provided by Cabot Microelectronics Corporation of Aurora, IL**.**
